# High performance sol–gel synthesized Ce_0.9_Sr_0.1_(Zr_0.53_Ti_0.47_)O_4_ sensing membrane for a solid-state pH sensor

**DOI:** 10.1039/c8ra03628d

**Published:** 2018-06-11

**Authors:** Sankar Prasad Bag, Prabir Garu, Jim-Long Her, Bih-Show Lou, Tung-Ming Pan

**Affiliations:** Department of Electronics Engineering, Chang Gung University Taoyuan 33302 Taiwan tmpan@mail.cgu.edu.tw +886-3-2118507 +886-3-2118800 ext. 3349; Division of Natural Science, Center for General Education, Chang Gung University Taoyuan 33302 Taiwan; Chemistry Division, Center for General Education, Chang Gung University Taoyuan 33302 Taiwan; Department of Nuclear Medicine and Molecular Imaging Center, Chang Gung Memorial Hospital Taoyuan 33302 Taiwan; Division of Urology, Chang Gung Memorial Hospital Taoyuan 33302 Taiwan

## Abstract

In this paper, we developed a high-performance solid-state pH sensor using a Ce_0.9_Sr_0.1_(Zr_0.53_Ti_0.47_)O_4_ (CSZT) membrane through a very simple sol–gel spin-coating process. The structural properties of the CSZT membrane are correlated with its sensing characteristics. The CSZT based EIS sensor exhibited a high pH sensitivity of 92.48 mV pH^−1^, which is beyond the Nernst limit (59.4 mV pH^−1^), and good reliability in terms of a low hysteresis voltage of 1 mV and a small drift rate of 0.15 mV h^−1^. This behaviour is attributed to the incorporation of Sr in the CSZT sensing membrane, which promotes change in the Ce oxidation state from Ce^4+^ to Ce^3+^.

## Abstract 2

We developed a high-performance solid-state pH sensor using a Ce_0.9_Sr_0.1_(Zr_0.53_Ti_0.47_)O_4_ (CSZT) membrane through a very simple sol–gel spin-coating process.

## Graphical abstract

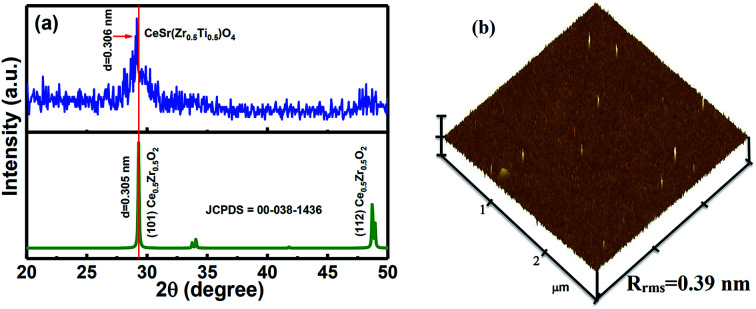

## Introduction

Determination of pH value is one of the most important measurements in all pH dependent chemical processes, especially in agricultural development, biochemical technology, the pharmaceutical industry, and corrosion control areas.^1^ Currently, the most often used electrode is a traditional glass-membrane pH electrode due to its Nernstian response and low sensitivity to interfering species.^2^ However glass electrodes have two main problems, namely the fragility of the glass membrane and easy fouling in aggressive electrolytes. Substantial progress has been recently made towards integrating high-*κ* dielectric materials into electrolyte–insulator–semiconductor (EIS) sensors due to their high sensing performance.^3–5^ EIS-based solid-state sensors, *e.g.* ion-sensitive field-effect transistors (ISFETs),^3^ light-addressable potentiometric sensors (LAPs),^6^ and organic thin-film transistors (OTFTs),^7^ have attracted intense interest in recent decades due to their simple structure, low cost, and easy fabrication process. The gate insulator plays the most important role in an EIS device because this insulating membrane is directly placed in an aqueous solution. The accumulated charges at the surface of the gate oxide film that arise from the electrolyte solution cannot be passed through the film, thus leading to the change in the channel conductance and current modulation.^4^

Different kinds of high-*κ* dielectric material, such as Al_2_O_3_, ZrO_2_, HfO_2_, Ta_2_O_5_, TiO_2_, and Y_2_O_3_,^5,8–11^ have been recently studied as sensing films in ISFET or EIS devices. Nevertheless, these materials can easily be reacted with Si substrates to form a silicate layer at the interface of the oxide film–Si substrate, thus degrading their electrical performance.^12^ To improve the electrical properties, rare-earth (RE) oxide film materials, including CeO_2_, Pr_2_O_3_, Gd_2_O_3_, and Yb_2_O_3_, have been investigated for use in complementary metal–oxide–semiconductor (CMOS), non-volatile memory, and EIS devices.^13–15^ However, moisture absorption is a major issue when RE oxides are used as gate dielectrics in CMOS devices, which degrades their electrical performance due to the formation of hydroxides.^16^ To avoid the unwanted hydroxide film, the Zr/Ti ratio of 0.53/0.47 was previously successfully adopted in a sensing film by our group.^17^ In this letter, we report the development of a high-performance Ce_0.9_Sr_0.1_(Zr_0.53_Ti_0.47_)O_4_ (CSZT) sensing membrane through a spin-coating process for a solid-state pH sensor, which is far beyond the Nernst limit of 59.4 mV pH^−1^ at 25 °C.

## Experimental

The CSZT mixed oxide membrane was synthesized with 1 N HNO_3_ and CH_3_COOH *via* a simple sol–gel method. The cerium acetate hydrate, strontium nitrate, zirconium propoxide, and titanium isopropoxide were mixed according to the molar ratio of Ce : Sr : Zr : Ti = 0.9 : 0.1 : 0.53 : 0.47. To adjust the concentration to 0.2 M with a total volume of 20 ml, acetic acid was used. After cleaning the 4-in p-type (100) Si wafer through a standard RCA process, the CSZT sensing membrane was deposited on a Si substrate using a spin-coating technique. After the spin-coating, the sample was placed on the hot plate at 150 °C for 5 min for solvent removal and then baked at 350 °C for 10 min for organic removal. To achieve good film quality, the membrane was annealed using a conventional furnace at 800 °C for 20 min in an oxygen atmosphere. A 100 nm-thick Al film was deposited on the backside of the wafer using a thermal evaporator to form good ohmic contact. To define the sensing area of the deposited CSZT, an automatic robotic dispenser was used through an adhesive silicone gel (S181) acting as a segregating layer. The EIS capacitive device was assembled on the Cu lines of a custom-made printed circuit board (PCB) by silver glue. To avoid the leakage from the electrolyte, an adhesive epoxy was deployed to encapsulate an EIS device and Cu. The pH sensitivity, hysteresis voltage, and drift rate of the CSZT EIS sensor were measured using an Agilent 4284A Precision LCR Meter with a Ag/AgCl reference and depicted with capacitance–voltage (*C*–*V*) curves. A reference electrode was employed to control and fix the potential between the electrolyte solution and EIS sensor.

## Results and discussion

**Fig. 1:**
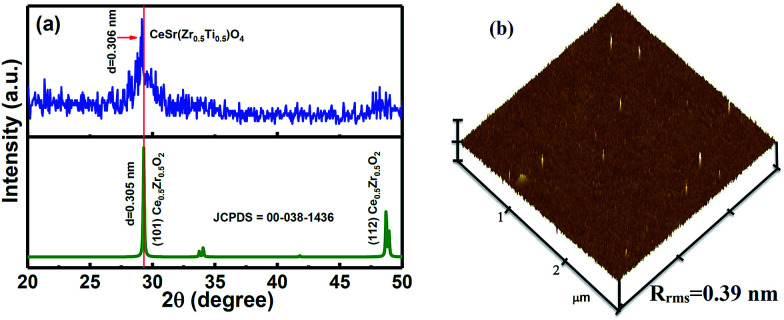
(a) XRD pattern and (b) AFM image of the CSZT membrane annealed at 800 °C for 30 min in an O_2_ atmosphere.

**Fig. 2:**
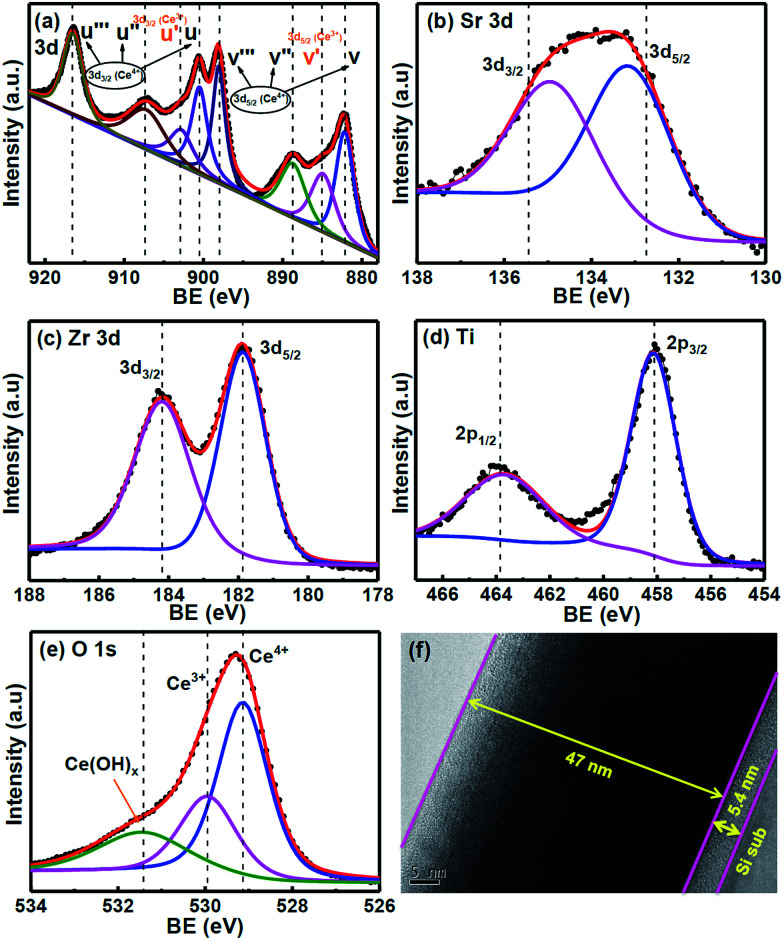
XPS spectra of (a) Ce 3d, (b) Sr 3d, (c) Zr 3d, (d) Ti 2p, and (e) O 1s of the CSZT membrane, and (f) HR-TEM image of the CSZT film.

Fig. 1(a) shows the X-ray diffraction (XRD) data of the CSZT membrane. The well-defined plane of (101) at 2*θ* = 29.15° is found in the XRD pattern, and is indicative of the fluorite-type tetragonal structure. In addition, the (101) peak position of the CSZT membrane was shifted to a lower 2*θ* value and the *d* spacing became higher (3.06 Å) relative to those of the Ce_0.5_Zr_0.5_O_2_ reference (JCPDS card no. 00-038-1436). This behaviour is mainly due to the higher ionic radii of the Ti and Sr incorporated into the CSZT membrane. Fig. 1(b) displays the atomic force microscopy (AFM) surface morphology image of the CSZT membrane. The surface roughness was estimated to be 0.39 nm. Fig. 2 displays the XPS spectra of (a) Ce 3d, (b) Sr 3d, (c) Zr 3d, (d) Ti 2p, and (e) O 1s for the CSZT membrane. The Ce 3d, Sr 3d, Zr 3d, Ti 2p, and O 1s element peaks were fitted using a combined symmetric Gaussian–Lorentzian line shape function after a Shirley background subtraction, except for the Ce 3d peak (linear background). Fig. 2(a) demonstrates that the Ce 3d spectra can be deconvoluted into eight peaks: v (882.6 eV), v′ (884.9 eV), v′′ (888.7 eV), v′′′ (898.1 eV), μ (900.4 eV), μ′ (902.7 eV), μ′′ (907.2 eV), and μ′′′ (916.5 eV). The four bands of μ and those of v represent Ce 3d_3/2_ and 3d_5/2_, respectively. The peaks of v and μ, v′′ and μ′′, and v′′′ and μ′′′ can be assigned to the (3d^9^4f^2^)O(2p^4^), (3d^9^4f^1^)O(2p^5^), and (3d^9^4f^0^)O(2p^6^) states of Ce^4+^, respectively, whereas the (3d^9^4f^1^)O(2p^6^) state of Ce^3+^ can be allotted to v′ and μ′.^18^ The ratio of Ce^3+^ to total Ce (namely, Ce^3+^ + Ce^4+^) is 21.36% higher than in previous studies [19.5% for Ce_2_(Zr_53_Ti_47_)O_4_ (CZT) film without Sr component].^17^ This is ascribed to the Sr incorporated into the CSZT membrane enhancing the change from Ce^4+^ to Ce^3+^ in the Ce oxidation state. Fig. 2(b) depicts that the Sr 3d_3/2_ and 3d_5/2_ double peaks at 134.8 eV and 133.1 eV, respectively, for the CSZT membrane are shifted toward higher binding energies compared with those of the SrTiO_3_ reference.^19^ The higher Sr 3d double binding energies of the CSZT film may be attributed to a mixture of Sr^2+^ ions in the CeO_2_ lattice. In addition, the ionic radius of Ce^4+^ (0.99 Å) is larger than that of Ti^4+^ (0.74 Å).^20^ Moreover, there is a shift in the Zr 3d_3/2_ and 3d_5/2_ split peaks (184.1 and 181.8 eV, respectively) to binding energies that are lower relative to those of ZrO_2_ film (185.8 and 182.2 eV, respectively),^21^ as shown in Fig. 2(c). The lower Zr 3d binding energy values for the CSZT film are presumably due to the alloy effect because Ce^4+^ is the predominant nearest neighboring cation. Fig. 2(d) shows that the Ti 2p_1/2_ and 2p_3/2_ split peaks located at 463.6 and 458.1 eV, respectively, are shifted toward binding energies that are lower relative to those of the TiO_2_ reference (464.3 and 458.7 eV, respectively),^22^ indicating the formation of Ce–Sr–O–Zr–Ti bonds. Fig. 2(e) demonstrates that the O 1s spectra of the CSZT membrane were convoluted with three appropriate curve-fitting lines. For the three spectra, the first peak at 531.4 eV represents Ce(OH)_*x*_, the second peak at 530 eV indicates the Ce^3+^ state, and the third peak at 529.1 eV indicates the Ce^4+^ state.^23,24^ The intensity of the O 1s peak corresponding to the Ce_2_O_3_ component is lower than that of CeO_2_, but is higher relative to our previous reports (the CZT film).^17^Fig. 2(f) shows the HR-TEM image of the CSZT membrane. The oxide thickness of the CSZT membrane was evaluated to be ∼47 nm.

**Fig. 3:**
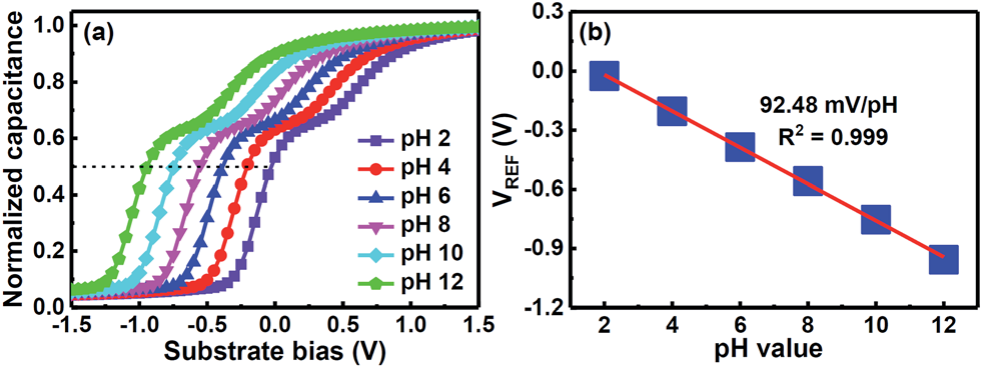
(a) Response of *C*–*V* curves and (b) reference voltage plotted with respect to pH at room temperature after inserting the CSZT-EIS sensor into the electrolyte with pH value ranging from 2 to 12.

Fig. 3(a) shows the *C*–*V* plots of the CSZT EIS sensor annealed at 800 °C for standard buffer solutions. To measure the sensitivity of the EIS device the shift in reference voltage (*V*_REF_), as shown in the *C*–*V* plots, was measured as it changes with the pH of the buffer solution due to mainly protonation or deprotonation which modifies the surface potential through dipole formation on the sensing membrane. Fig. 3(b) presents the *V*_REF_ of the CSZT EIS sensor as a function of pH. The CSZT EIS sensor exhibited a super-Nernstian pH sensitivity of 92.48 mV pH^−1^ with a linear response in the range of pH 2–12, which is far larger than the theoretical Nernstian value (59.4 mV pH^−1^ at 25 °C). This super-Nernstian response may be attributed to the incorporation of Sr into the CSZT film, which enhances the change in the Ce oxidation state from Ce^4+^ to Ce^3+^. In this case, we could suspect that in a mild solution, the oxidized Ce^4+^ ion and reduced Ce^3+^ ion participate in the redox reactions below:

1 Ce(OH)_3_ ↔ Ce(OH)_2_O^−^ + H^+^

2 Ce_2_O(OH)_6_ ↔ Ce_2_O(OH)_2_O_4_^4−^ + 4H^+^

3 2Ce(OH)_3_ + H_2_O ↔ Ce_2_O(OH)_6_ + 2H^+^ + 2e^−^

Substituting eqn (1) and (2) into (3), we can obtain the stoichiometric redox reaction:

4 2Ce(OH)_2_O^−^ + H_2_O ↔ Ce_2_O(OH)_2_O_4_^4−^ + 4H^+^ + 2e^−^

From this reaction, only two electrons are transferred per four protons (proton/electron ratio of 2), hence a sensitivity of 118.8 mV pH^−1^ was achieved because of the mixing of the oxidized CeO_2_ and reduced Ce_2_O_3_ states in the CSZT membrane. The empirical result of the pH sensitivity being over 59.4 mV pH^−1^ can be explained by there being less than one electron per proton transferred in the redox reaction. In contrast, the pH sensitivity being below 59.4 mV pH^−1^ might be due to there being more than one electron per proton transferred in this reaction.

**Fig. 4:**
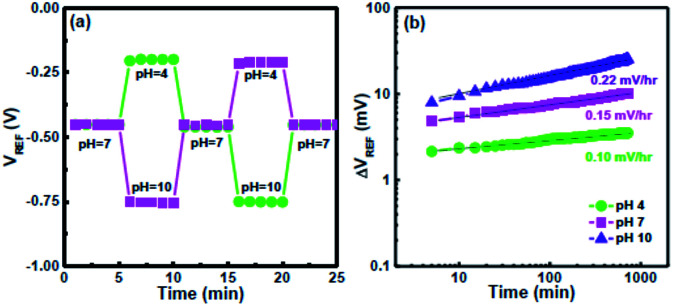
(a) Hysteresis curves of the CSZT EIS sensor measured in the pH loops 7 → 4 → 7 → 10 → 7 and 7 → 10 → 7 → 4 → 7. (b) Drift characteristics of the CSZT EIS sensor tested at pH 4, 7 and 10.

To evaluate the hysteresis of the CSZT EIS sensor, it was subjected to two pH loops of 7 → 4 → 7 → 10 → 7 and 7 → 10 → 7 → 4 → 7 over a period of 1500 s, as shown in Fig. 4(a). The hysteresis voltages were estimated to be 1 and 1.5 mV for the 7 → 4 → 7 → 10 → 7 and 7 → 10 → 7 → 4 → 7 loops, respectively. The smaller hysteresis of the CSZT film is attributed to the formation of a CSZT stoichiometric structure, thus leading to minimal buried oxide. Fig. 4(b) demonstrates the drift rate of an EIS sensor measured at pH 4, 7 and 10 for 12 h. The CSZT EIS sensor exhibited a low drift rate of 0.15 mV h^−1^ at pH 7. In this study, our CSZT membrane demonstrated a pH detection sensitivity (92.48 mV pH^−1^) that is superior to those of materials commonly used for EIS or ISFET-based sensors, such as Al_2_O_3_ (58 mV pH^−1^),^8^ TiO_2_ (55 mV pH^−1^),^5^ Ta_2_O_5_ (56–58 mV pH^−1^),^8^ ZrO_2_ (∼55 mV pH^−1^),^9^ HfO_2_ (∼55 mV pH^−1^),^9^ Y_2_O_3_ (54.5 mV pH^−1^),^11^ and CZT (64.42 mV pH^−1^).^17^

## Conclusions

In summary, we have demonstrated a high-performance CSZT membrane deposited on a Si substrate through a simple sol–gel spin-coating process. A high pH sensitivity of 92.48 mV pH^−1^, a small hysteresis voltage of 1 mV, and a low drift rate of 0.15 mV h^−1^ were achieved by the CSZT EIS sensor. These results are attributed to the incorporation of Sr in the CSZT, which enhances the change in the Ce oxidation state from Ce^4+^ to Ce^3+^, resulting in a rise in the ratio of protons to electrons transferred in the redox reaction. This CSZT membrane EIS sensor can be used in future solid-state biosensor devices.

## Conflicts of interest

There no conflicts to declare.

## Supplementary Material

## References

[cit1] Janata J. , Principles of Chemical Sensors, Plenum Press, New York, 1990

[cit2] Glass Electrodes for Hydrogen and Other Cations, ed. G. Eisenman, Marcel Dekker, New York, 1967

[cit3] Bergveld P. (1970). IEEE Trans. Biomed. Eng..

[cit4] Schoning M. J., Poghossian A. (2006). Electroanalysis.

[cit5] Fog A., Buck R. P. (1984). Sens. Actuators.

[cit6] Yoshinobu T., Schoning M. J., Otto R., Furuichi K., Mourzina Y., Ermolenko Y., Iwasaki H. (2003). Sens. Actuators, B.

[cit7] Tanese M. C., Fine D., Dodabalapur A., Torsi L. (2005). Biosens. Bioelectron..

[cit8] Matsuo T., Esashi M., Abe H. (1979). IEEE Trans. Electron Devices.

[cit9] Jankovic V., Chang J. P. (2011). J. Electrochem. Soc..

[cit10] Schoning M. J., Brinkmann D., Rolka D., Demuth C., Poghossian A. (2005). Sens. Actuators, B.

[cit11] Pan T. M., Liao K. M. (2007). Sens. Actuators, B.

[cit12] Houssa M. , High-k Gate Dielectrics, Institute of Physics Publishing, Bristol and Philadelphia, 2004

[cit13] Fanciulli M. and Scarel G., Rare Earth Oxide Thin Film: Growth, Characterization, and Applications, Springer, Berlin, 2007

[cit14] Ismail M., Rana A. M., Talib I., Tsai T. L., Chand U., Ahmed E., Younus Nadeem M., Aziz A., Shah N. A., Hussain M. (2015). Solid State Commun..

[cit15] Pan T. M., Huang W. S. (2009). J. Electrochem. Soc..

[cit16] Jeon S., Hwang H. (2003). J. Appl. Phys..

[cit17] Bag S. P., Lou B. S., Her J. L., Pang S. T., Pan T. M. (2017). IEEE Trans. Electron Devices.

[cit18] Dauscher A., Hilaire L., Le Normand F., Miiller W., Maire G., Vasqwz A. (1990). Surf. Inter. Ana..

[cit19] Zhang L., Tian W., Chen Y., Chen J., Teng H., Zhou J., Shi J., Sun Y. (2016). RSC Adv..

[cit20] Gupta A., Kumar A., Waghmare U. V., Hegde M. S. (2009). Chem. Mater..

[cit21] Takahashi N., Yoshii N., Nonobe S., Nakamura T., Yoshioka M. (2003). J. Electron. Mater..

[cit22] Kang B. S., Sul Y. T., Oh S. J., Lee H. J., Albrektsson T. (2009). Acta Biomater..

[cit23] Sinha A. K., Suzuki K. (2005). J. Phys. Chem. B.

[cit24] Stetsovych V., Pagliuca F., Dvorak F., Duchon T., Vorokhta M., Aulicka M., Lachnitt J., Schernich S., Matolínova I., Veltruska K., Skala T., Mazur D., Myslivecek J., Libuda J., Matolin V. (2013). J. Phys. Chem. Lett..

